# Resolving candidate genes of mouse skeletal muscle QTL via RNA-Seq and expression network analyses

**DOI:** 10.1186/1471-2164-13-592

**Published:** 2012-11-05

**Authors:** Arimantas Lionikas, Caroline Meharg, Jonathan MJ Derry, Aivaras Ratkevicius, Andrew M Carroll, David J Vandenbergh, David A Blizard

**Affiliations:** 1 School of Medical Sciences, University of Aberdeen, Aberdeen, AB25 2ZD, UK; 2 School of Medicine & Dentistry, University of Aberdeen, Aberdeen, AB25 2ZD, UK; 3 Bioinformatics group, Max Planck Institute for Biology of Aging, Köln, D-50931, Germany; 4 Sage Bionetworks, Seattle, WA, 98109, USA; 5 Department of Biobehavioral Health, University Park, PA, 16802, USA; 6 Penn State Institute for the Neurosciences, University Park, PA, 16802, USA; 7 College of Health and Human Development, The Pennsylvania State University, University Park, PA, 16802, USA

**Keywords:** Functional genomics, QTL, Skeletal muscle, Gene expression

## Abstract

**Background:**

We have recently identified a number of Quantitative Trait Loci (QTL) contributing to the 2-fold muscle weight difference between the LG/J and SM/J mouse strains and refined their confidence intervals. To facilitate nomination of the candidate genes responsible for these differences we examined the transcriptome of the tibialis anterior (TA) muscle of each strain by RNA-Seq.

**Results:**

13,726 genes were expressed in mouse skeletal muscle. Intersection of a set of 1061 differentially expressed transcripts with a mouse muscle Bayesian Network identified a coherent set of differentially expressed genes that we term the LG/J and SM/J Regulatory Network (LSRN). The integration of the QTL, transcriptome and the network analyses identified eight key drivers of the LSRN (*Kdr*, *Plbd1*, *Mgp*, *Fah*, *Prss23*, *2310014F06Rik*, *Grtp1*, *Stk10*) residing within five QTL regions, which were either polymorphic or differentially expressed between the two strains and are strong candidates for quantitative trait genes (QTGs) underlying muscle mass. The insight gained from network analysis including the ability to make testable predictions is illustrated by annotating the LSRN with knowledge-based signatures and showing that the SM/J state of the network corresponds to a more oxidative state. We validated this prediction by NADH tetrazolium reductase staining in the TA muscle revealing higher oxidative potential of the SM/J compared to the LG/J strain (p<0.03).

**Conclusion:**

Thus, integration of fine resolution QTL mapping, RNA-Seq transcriptome information and mouse muscle Bayesian Network analysis provides a novel and unbiased strategy for nomination of muscle QTGs.

## Background

The aim of functional genomics is to understand the role of specific genes in phenotypic variation. The *forward genetics* approach has led to a large number of identified genomic regions, known as quantitative trait loci (QTL), influencing various phenotypes, including those for muscle weight [1-4]. However, a bottle neck has developed in the transition from QTL to their causative quantitative trait genes (QTG) [5]. Advanced intercross line strategy permits accumulation of recombinations and improves resolution of QTL mapping [6], which in the case of muscle weight has led to major reduction in confidence intervals [1]. Although appreciably refined, these QTL still harbour a number of genes. Thus, further efforts are needed to identify the QTGs that are the causative factors in complex traits.

It has been proposed that testing for the expression differences could identify genes underlying phenotypic differences [7]. Implementation of such strategy led to several nominations of QTG’s [8,9]. However, microarray technology, used as a tool for a high throughput expression analyses, has several limitations which might have interfered with a more productive contribution of this approach to the nomination of the candidate genes. Hybridization artefacts caused by SNP’s [10], non linearity among probes, inability to detect splice variants and, importantly, the bias towards available information (i.e. only transcripts with corresponding probes on microarray can be examined), limit the utility of expression microarrays. Transcriptome analyses by means of a massive parallel sequencing technology, RNA-Seq, circumvents the above-mentioned limitations [11,12], it is highly replicable [13] and therefore a very attractive research method for an unbiased identification of differentially expressed genes.

Our QTL studies focused on muscle size, which is an important variable influencing health and quality of life particularly in the elderly which are affected by sarcopenia, age-related muscle wasting [14]. In addition, skeletal muscle tissue is a major component of diet and a source of nutrients for the growing population of the planet. Genetic variation plays a substantial role in determining muscle size in mammals [1-4,15,16] but the underlying genes remain largely unknown. Muscle mass is a function of the number and size of its fibers. The number of fibers in mouse is determined prenatally and remains stable throughout adulthood [17], whereas cross sectional area (CSA) of the fibres increases during post-natal development [18].

The LG/J and SM/J strains, which were selected for large and small body weight, respectively, in order to study processes related to growth [19,20], is a promising model system for exploration of the genetic effects on muscle mass. These strains differ prominently in mass of several hind limb muscles (2-fold difference between them) and 22 QTL contributing to this difference were mapped [1]. Subsequent analyses of the soleus muscle found that the number of fibres in the muscle of the two strains was similar, whereas CSA differed substantially, LG/J > SM/J [21].

The phenotypic differences due to genetic variation are determined by the pattern of information flow through molecular networks [22]. A mouse muscle Bayesian Network (MMBN) has been recently constructed based on genetic and gene expression data. The MMBN provides directional information about the relationship of gene expression and can be used as a tool for identification of the key drivers of gene expression signatures characterising various phenotypes [23].

The main finding of the present study is that the integration of the gene expression signature with the QTL analysis and muscle gene network data can lead to the identification of plausible QTGs underlying phenotypic differences in muscle mass.

## Results

### Muscle weight

The tibialis anterior (TA) muscle weight (mean of the right and left hindlimb) was 62.4 ± 2.5 mg *vs* 37.2 ± 1.5 mg for males of LG/J and SM/J strains, respectively (strain effect p<0.001), and 50.2 ± 1.8 mg *vs* 28.0 ± 1.7 mg (p<0.0001) for females. These estimates are comparable with our earlier findings at the same age [1]. A similar degree of the strain difference was observed in EDL, gastroncemius and soleus muscles [1].

### Global transcriptome

Of 36,536 genes on the reference genome mm9, reads mapped to 22,630 genes (Additional file 1). To establish a threshold for the reliable detection of gene expression, we analysed Y chromosome genes in the female samples. From the Deseq analysis we noted that reads mapped to 5 out of 14 Y chromosome genes in females of at least one strain with the highest expression value of 8.1 for *Eif2s3y* gene. Because expression of this gene is much more robust in male samples, 323.3, we excluded possibility of sample mixup. Therefore, we considered that values equal or below the 8.1 threshold were not reliable. Only the transcripts exceeding it in at least one strain were flagged as expressed in skeletal muscle (Additional file 1). There were 13,726 such genes and their transcripts accounted for >99.9% of the mapped transcriptome. Expression levels differed greatly among the genes identified by these RNA-seq tests (Additional file 2); the 75 most abundantly expressed genes accounted for approximately the same fraction of the transcriptome as all remaining genes.

The differential expression of genes between the LG/J and SM/J strains was assessed using 3 different statistical methods. The most conservative method, Bayesian Negative-Binomial-Method-Likelihood normalization (Bayesian-NBML), found 577 differentially expressed genes at false discovery rate (FDR) of p<0.1. The most liberal method, Bayesian-Poisson normalization, led to 9,086 genes at p<0.1, whereas the Deseq procedure resulted in 1,184 genes at p<0.1. A list of 1061 differentially expressed (DE) genes were identified by two out of three procedures. The DE genes were subjected to various further analyses.

### QTL – expression cross-reference

A previous study [1] found that muscle weight differences between the LG/J and SM/J strains are affected by 22 QTL (loci *Skmw21* - *Skmw42*) located on chromosomes 2, 4, 5, 6, 7, 8 and 11. The cumulative genomic length of the loci was 75.5 Mb. The overlap between a gene’s nomination by its presence under a QTL, and its differential expression was analysed next.

#### Expression differences

There were 1099 genes within the 22 QTL regions. Nearly half of them, 459, were expressed in TA muscle which is a 1.1-fold enrichment (hypergeometric p value= 0.00037) of expressed genes within the QTL regions. We then cross-referenced this list of genes with 1061 DE genes between the LG/J and SM/J strains (Additional file 1). This analysis identified 41 DE genes within eighteen QTL (Table 1). It represents a 1.3-fold enrichment of DE genes within QTL regions (hypergeometric p value = 0.018).

**Table 1:** **The candidate genes nominated by expression difference and**/**or coding polymorphisms for skeletal muscle weight QTL** (***Skmw***) **affecting muscle weight between the LG**/**J** (**L**) **and SM**/**J** (**S**) **strains**

**Gene**	**Chr**	**QTL**	**Type**
*BC029722*	2	Skmw21	L < S
*Uqcc*	2	Skmw21	L > S
*Adig*	2	Skmw22	L < S
*Ppp1r16b*	2	Skmw22	L < S
*Fam83d*	2	Skmw22	L > S
*Gm826*	2	Skmw22	L < S
*Chd6*	2	Skmw22	L > S
*Rasl11b*	5	Skmw24	L < S
*Kdr*	5	Skmw24	L < S **L1176F**
*Ppat*	5	Skmw24	L > S
*Osbpl3*	6	Skmw25	L < S
*Mrpl19*	6	Skmw26	L < S
*Htra2*	6	Skmw26	T449D
*Adamts9*	6	Skmw27	P699A
*Gm15737*	6	Skmw27	L < S
*Rybp*	6	Skmw28	L > S
*Il17re*	6	Skmw29	L > S
*Irak2*	6	Skmw29	L > S
*Sec13*	6	Skmw29	K87M
*Plbd1*	6	Skmw31	L < S
*Mgp*	6	Skmw31	L<S **Q55L**
*Rerg*	6	Skmw31	L < S
*Synm*	7	Skmw32	R946Q **P835S**
*Pgpep1l*	7	Skmw32	L < S
*Mesdc2*	7	Skmw33	L < S
*Fah*	7	Skmw33	L > S
*Nox4*	7	Skmw33	L < S
*Prss23*	7	Skmw33	L > S
*Mical2*	7	Skmw34	L > S
*2310014F06Rik*	7	Skmw34	L < S
*Arntl*	7	Skmw34	L > S
*Fgfr2*	7	Skmw35	L > S
*Irs2*	8	Skmw36	L < S
*Col4a1*	8	Skmw36	L < S
*Col4a2*	8	Skmw36	L < S
*Lamp1*	8	Skmw36	R294H
*Grtp1*	8	Skmw36	L < S
*Adprhl1*	8	Skmw36	L > S
*Tfdp1*	8	Skmw36	R302H
*2610019F03Rik*	8	Skmw36	L > S
*Snord13*	8	Skmw37	L > S
*Dlc1*	8	Skmw37	L < S
*Lphn1*	8	Skmw39	L > S
*Podnl1*	8	Skmw39	L > S
*Adcy1*	11	Skmw40	L > S
*Tns3*	11	Skmw40	**T1067M**
*Rhbdf1*	11	Skmw42	R42W
*Hba*-*a2*	11	Skmw42	L > S
*Stk10*	11	Skmw42	L < S

Type indicates whether gene is more (>) or less (<) abundant, or position and swapped amino acids (substitutions in bold are more likely to affect protein function than those in regular font).

**Figure 1:**
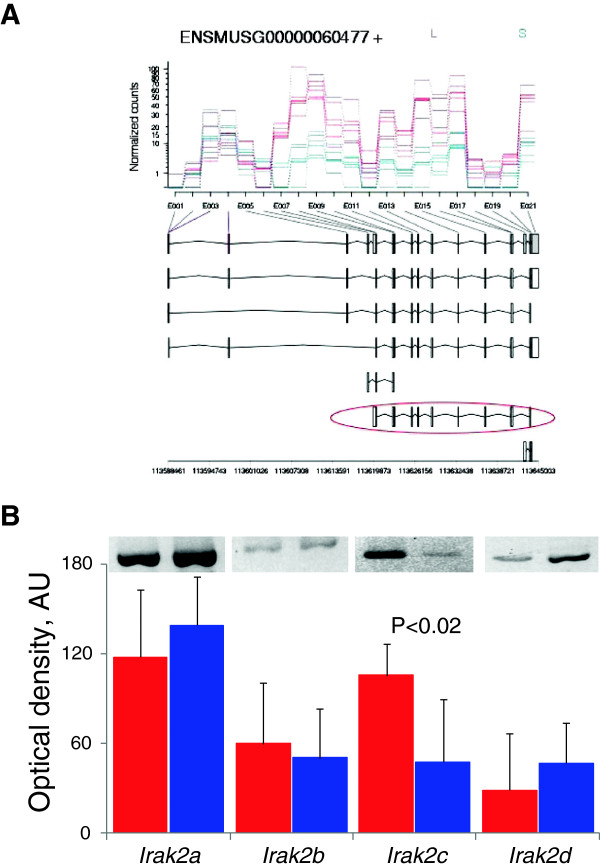
**Splice variants of *Irak2 *gene in TA muscle from LG**/**J and SM**/**J strains.** (**A**) Dexseq analysis identified differential expression of a number of exons between the LG/J (red line) and SM/J (blue line) strains. The pattern of differential expression overlapped with ENSMUST00000113024 transcript (outlined in red at the bottom) which corresponds to *Irak2c* splice variant [24]. (**B**) Abundance of the splice-specific PCR amplicones was compared between the strains (red – LG/J, blue – SM/J). Mean ± SD of six samples per strain (representative gels presented above corresponding bars).

Analysis with DexSeq identified 319 differentially expressed exons at FDR<0.1 (Additional file 3), nine genes with such exons resided within QTL regions. Of those, expression of the *Irak2* gene appeared to be higher in the LG/J strains across a number of exons (Table 2). To verify the prediction of the presence of different splice variants we examined the expression levels of the four reported splice variants. Transcript ENSMUST00000113024, which corresponds to the characterised spice variant *Irak2c*[24], was overrepresented in the LG/J strain compared to SM/J, whereas the other examined variants did not differ between the strains (Figure 1).

**Table 2:** **Exon**-**specific DexSeq analysis of expression of *Irak2 *gene **

**DexSeq-exonID**	**Irak2 exon**	**Normalised Mean Count comparison**				**DexSeq Statistical Results**				
		**LG/J mean**	**SM/J mean**	**log2fold (S/L) Calculated from normlalised mean counts**	**P value**	**Disp Before Sharing**	**Disp Fitted**	**dispersion**	**log2 fold (S/L) DexSeq calculated**	**padjust**
E001	1	0.21	0	0	0.3632	NA	NA	NA	NA	NA
E002	1	1.26	1	0	0.6956	0	2.04	2.04	2.13	1
E003	1	7.24	9.28	0.35	0.5647	0.4	0.2	0.4	2.83	0
E004	2	13.77	11.67	−0.02	0.9647	0.09	0.2	0.2	2.54	0
E005	3	6.33	3.3	−0.57	0.3403	0.17	0.48	0.48	2	0.8
E006	4	0.82	2.22	1.24	0.0819	0.47	1.41	1.41	3.75	0.76
E007	4	**10**.**59**	**0**.**49**	−**4**.**03**	**0**.**0001**	0.17	0.31	0.31	−1.75	1
E008	4	**45**.**28**	**3**.**16**	−**3**.**48**	**0**.**0067**	0.09	0.13	0.13	−0.91	1
E009	5	**51**.**11**	**8**.**5**	−**2**.**67**	**0**.**0002**	0.05	0.11	0.11	−0.24	1
E010	5	**27**.**25**	**3**.**97**	−**2**.**53**	**0**.**0198**	0.16	0.19	0.19	−0.14	1
E011	6	**18**.**98**	**1**.**62**	−**2**.**94**	**0**.**017**	0.12	0.23	0.23	−0.51	1
E012	7	**2**.**67**	**0**.**24**	−**1**.**42**	**0**.**0424**	0.3	1.59	1.59	−1.57	1
E013	8	**17**.**32**	**2**.**33**	−**2**.**83**	**0**.**0019**	0.18	0.21	0.21	−0.32	1
E014	9	**11**.**27**	**0**.**62**	−**3**.**54**	**0**.**0041**	0	0.41	0.41	−1.42	1
E015	10	**39**.**58**	**2**.**95**	−**3**.**65**	**0**.**0009**	0.06	0.14	0.14	−1.18	0.69
E016	11	**31**.**23**	**4**.**41**	−**2**.**84**	**0**.**017**	0.28	0.18	0.28	−0.41	1
E017	12	**48**.**16**	**9**.**62**	−**2**.**4**	**0**.**001**	0.03	0.12	0.12	0.03	1
E018	12	1.25	1.22	−1	0.2566	1.07	1.59	1.59	1.1	1
E019	13	0.8	0.49	0	0.4139	0.14	3.09	3.09	1.38	1
E020	13	**3**.**13**	**0**.**24**	−**2**.**01**	**0**.**0004**	0	1.07	1.07	−1.27	1
E021	13	**39**.**8**	**4**.**41**	−**2**.**46**	**0**.**0021**	0.16	0.13	0.16	0.06	1

The pattern of exons with the higher expression of normalised counts in LG/J samples (bold) corresponds to *Irak2c* splice variant. Irak2 foldchanges calculated from normalised mean counts show overexpression of exon-bins 7–17, 20 and 21 in the *Irak2* gene model which standard DexSeq analysis failed to detect.

DexSeq-exonID – exon bins used by DexSeq; LG/J mean and SM/J mean – normalized count of reads per bin; *Irak2* exons – Ensembl based exons, some exons were partitioned into several DexSeq bins; P-values – nominal p value of t-test between the LG/J and SM/J samples.

#### Polymorphisms

The allelic effect could be brought about by a different level of gene expression or by altered coding sequence. Therefore, it was important to examine the coding polymorphisms captured in the transcriptome of the two strains. A total of 7,933 (673 missense) SNPs (Additional file 4) and 150 Indels (Additional file 5) were identified between the LG/J and SM/J strains.

In the subsequent analysis we focused only on the genes residing within the *Skmw* loci [1] . First, the Indel data were examined revealing a three nucleotide insertion (CTT) in exon 5 of alpha-kinase 3 gene, *Alpk3*, of the LG/J strain. Although the gene is not within the reported boundaries (which were determined as the region overlapped between the QTL of first principal component extracted from 5 different muscles and EDL weight QTL) of the *Skmw33* locus (1.9 Mb proximal from the centromeric boundary) it is well within the QTL affecting weight of the EDL muscle [1].

We then analyzed the non-synonymous SNPs with the PolyPhen tool which predicts possible impact of an amino acid substitution on the structure and function of the protein. This analysis revealed that substitution of amino acids in 10 genes residing within the *Skmw* loci might affect the function of the proteins (Table 1). Two of those genes, *Mgp* and *Kdr*, were also differentially expressed between the strains (Additional file 1).

### Network Analysis

In addition to using differential expression and/or sequence differences to prioritize QTL region genes we took advantage of an independent mouse muscle Bayesian Network to identify putative regulators. The network was constructed from gene expression and genetic data from nine different mouse F2 crosses encompassing >3,000 mice and nine different mouse strains. The construction of mouse F2 Bayesian networks from genetic and expression data has been described elsewhere [25,26]. For this analysis we used muscle tissue networks constructed from nine different mouse F2 crosses including: BTBR ob/ob x B6 ob/ob (BTBRxB6ob) [27], C57BL/6J x C3H/HeJ (Bxh) [28], C57BL/6J Apoe−/− x C3H/HeJ Apoe−/− (BxHapoe) [29], C57BL/6J x A/J (Bxa JaxS) [30], C57BL/6J x 129S1/SvImJ (Bx129_JaxS), C57BL/6J x DBA/2J (BxD JaxS), C57BL/6J x DBA/2J (BxD JaxL), C57BL/6J x A/J (BxA MCI) [30] and C57BL/6J x DBA/2J (BxD PSU) [2]. We constructed both gender specific networks and combined networks where possible to generate a complete set of interactions. For the purposes of analysis we created a union of the 18 individual networks and included only nodes for which probes could be clearly mapped to high confidence genes. The resulting Mouse Muscle Bayesian Network (MMBN) consists of 19,513 individual nodes, corresponding to genes, and 75,092 edges, corresponding to associations between genes (Additional File 6).

First we explored the distribution of DE genes between the LG/J and SM/J strains within the network. A total of 855 DE genes mapped within the MMBN and remarkably, 405 genes mapped within a single coherent network exclusively containing differentially expressed genes (genes belonging to these networks are flagged in Additional File 1). This is highly significant (p<0.001) - we did not detect a similarly sized or larger coherent network with 1,000 randomly selected gene sets of the same size – and suggests that a large portion of the genes that are differentially expressed between the LG/J and SM/J strains are co-ordinately regulated in mouse muscle independent of strain.

The Bayesian network is a directed network and hence can be used to predict regulators of a particular signature or gene set [31]. An algorithm has been recently developed, called Key Driver Analysis [23] that can be used to search a network for genes whose downstream children are enriched in genes of a signature of interest. We took the signature corresponding to the LG/SM DE genes that are contained within the network (n=855) and ran the key driver analysis, thereby identifying 3,556 putative key drivers. Cross-reference of the 3,556 putative drivers of the differential signature with the 1099 genes in the QTL regions identified 116 genes (Additional file 7). This represents a significant 1.5-fold enrichment of the key drivers within the QTL regions (hypergeometric p value=2.29E-6).

**Figure 2:**
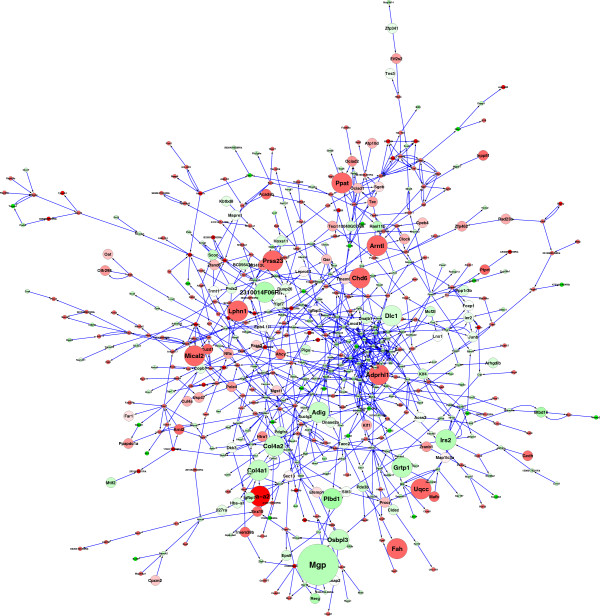
**The 251 node network is a sub**-**network extracted from a large mouse muscle Bayesian network of>19**,**500 nodes using a LG**/**J v SM**/**J differentially expressed gene set of 781 genes and their putative regulators.** The nodes are colored according to differential expression; red=up regulated in LG/J *vs* SM/J; green = down-regulated in LG/J *vs* SM/J. Larger nodes are matching three out of four criteria: contained in QTL critical region; predicted key driver; significantly differentially expressed; amino acid change between strains.

When combined with the 49 genes identified by differential expression or polymorphism (Table 1) this generates a non-redundant list of 142 putative regulators. Eighteen of these are contained within the 405 gene coherent network and a further 80 are within 1-edge. Figure 2 shows a 545 gene network that contains the 405 DE genes and their putative regulators; we refer to this as the LG/J and SM/J Regulatory Network (LSRN). Eight of 116 key drivers (*Kdr*, *Plbd1*, *Mgp*, *Fah*, *Prss23*, *2310014F06Rik*, *Grtp1*, *Stk10*) residing within five QTL regions were either polymorphic or differentially expressed between the LG/J and SM/J strains and therefore are strong candidates to explain the effects of these loci.

To explore properties of the LSRN further we carried out gene annotation enrichment analysis using two online bioinformatics tools; DAVID [32] and NextBio [32]. DAVID revealed a significant enrichment for GO-Terms relevant to muscle structure, function and bioenergetics. Indeed these GO-terms are very similar to those obtained with the full DE gene set (Table 3) suggesting that the LSRN is a relevant subset of DE genes. A complete set of DAVID analysis results is presented in Additional File 8. Aside from GO-terms related to muscle structural components analysis of the LSRN and DE genes identified a number of mitochondrial gene set enrichments suggesting potential metabolic difference between the LG/J and SM/J muscles. We explored this further by mining a larger set of internally curated signatures as well as those from NextBio. The most significant experiments identified through this analysis included signatures of expression in mouse quadriceps muscle subjected to AMPK and PPARδ agonists [33,34] and in gastrocnemius of mice subjected to hindlimb suspension [35]; 11.2-fold, p=3.25e-11 and 2.2-fold, p=4.7e-17 respectively enrichment in the LSRN relative to the whole network. These treatments are known to have profound effects on the metabolic state of the muscle leading us to bolster our hypothesis that the differences between LG/J and SM/J may at least in part be due to fundamental metabolic variation; overlay of the expression differences with the TA muscle of the LG/J and SM/J mice strongly predicts that the SM/J strain has a more oxidative profile than the LG/J strain.

**Table 3:** **Gene ontology** (**GO**) **terms enriched in LSRN and DE sets of genes expressed in LG**/**J and SM**/**J strain tibialis anterior muscle**

**GO**-**Terms enriched**			**DE genes**					**LSRN genes**				
**Category**	**Go**-**Term**	**Go**-**Description**	**Count**	**%**	**PValue**	**Fold Enrichment**	**Benjamini**	**Count**	**%**	**PValue**	**Fold Enrichment**	**Benjamini**
GOTERM_CC_FAT	GO:0043292	contractile fiber	18	1.83	1.87E-06	4.01	9.10E-05	16	3.02	7.06E-08	5.88	6.99E-06
GOTERM_CC_FAT	GO:0044449	contractile fiber part	17	1.73	2.20E-06	4.18	9.36E-05	15	2.84	1.31E-07	6.09	9.70E-06
GOTERM_CC_FAT	GO:0030016	myofibril	18	1.83	9.98E-07	4.18	5.67E-05	16	3.02	3.89E-08	6.14	1.16E-05
GOTERM_CC_FAT	GO:0044429	mitochondrial part	49	4.99	7.64E-06	1.98	2.61E-04	36	6.81	2.55E-06	2.40	1.51E-04
GOTERM_CC_FAT	GO:0016460	myosin II complex	6	0.61	1.38E-04	10.58	3.36E-03	6	1.13	1.24E-05	17.46	6.13E-04
GOTERM_CC_FAT	GO:0005759	mitochondrial matrix	21	2.14	8.33E-05	2.73	2.36E-03	17	3.21	1.70E-05	3.64	7.23E-04
GOTERM_CC_FAT	GO:0031980	mitochondrial lumen	21	2.14	8.33E-05	2.73	2.36E-03	17	3.21	1.70E-05	3.64	7.23E-04
GOTERM_CC_FAT	GO:0032982	myosin filament	6	0.61	4.65E-04	8.46	9.87E-03	6	1.13	4.37E-05	13.97	1.62E-03
GOTERM_CC_FAT	GO:0016459	myosin complex	11	1.12	5.18E-04	3.82	1.03E-02	10	1.89	5.33E-05	5.73	1.76E-03
GOTERM_CC_FAT	GO:0005859	muscle myosin complex	5	0.51	8.20E-04	10.58	1.26E-02	5	0.95	1.20E-04	17.46	2.73E-03
GOTERM_CC_FAT	GO:0005739	mitochondrion	94	9.58	4.62E-05	1.50	1.43E-03	62	11.72	1.02E-04	1.64	2.75E-03
GOTERM_CC_FAT	GO:0005740	mitochondrial envelope	34	3.47	8.90E-04	1.84	1.31E-02	24	4.54	8.65E-04	2.14	1.50E-02
GOTERM_MF_FAT	GO:0017076	purine nucleotide binding	117	11.93	1.90E-04	1.38	4.57E-02	80	15.12	6.56E-05	1.54	1.85E-02
GOTERM_CC_FAT	GO:0005863	striated muscle thick filament	4	0.41	4.90E-03	10.58	6.24E-02	4	0.76	1.16E-03	17.46	1.90E-02
GOTERM_CC_FAT	GO:0044421	extracellular region part	65	6.63	6.92E-06	1.78	2.62E-04	38	7.18	1.42E-03	1.71	2.20E-02
GOTERM_CC_FAT	GO:0005743	mitochondrial inner membrane	28	2.85	8.09E-04	2.00	1.31E-02	19	3.59	2.12E-03	2.24	2.96E-02
GOTERM_CC_FAT	GO:0031966	mitochondrial membrane	31	3.16	2.53E-03	1.78	3.39E-02	22	4.16	2.04E-03	2.09	2.98E-02

Count – number of genes from the list involved in term; % - percentage of all genes in the list; PValue – modified Fisher Exact p-Value for enrichment; Benjamini – false discovery rate corrected P-values.

**Figure 3:**
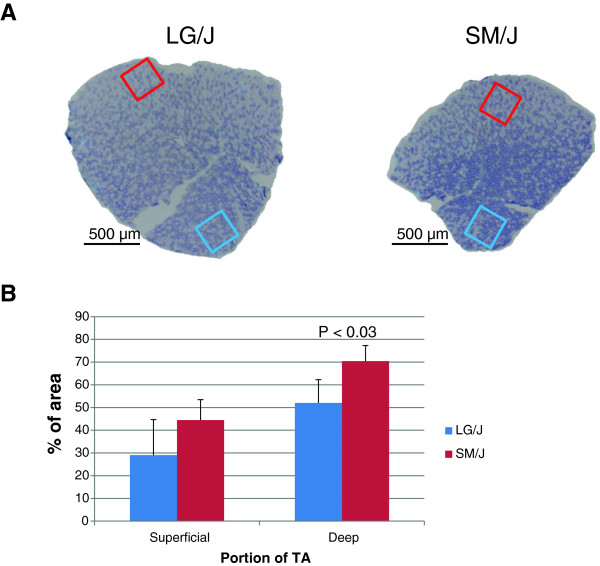
**NADH tetrazolium reductase staining in TA muscle.** Representative areas of the superficial (panel **A**, red box) and deep (blue box) portion of the muscle were quantified for the staining intensity (darker stain corresponds to a higher oxidative capacity). Panel **B**; mean and SD (n=4 per strain) of the area occupied by the dark stained, oxidative, fibers.

This prediction was tested and confirmed by NADH tetrazolium reductase staining (Figure 3). The TA of the SM/J strain exhibited greater oxidative potential particularly in the deep portion of the muscle (p<0.03).

## Discussion

We integrated a mouse muscle Bayesian Network and transcriptome data from the muscle of two inbred strains, LG/J and SM/J, with the results of QTL mapping of muscle weights in an advanced intercross [1] to nominate genes contributing to a 2-fold difference in muscle mass. The analyses based on three independent sources of information converged on a set of eight genes (*Kdr*, *Plbd1*, *Mgp*, *Fah*, *Prss23*, *2310014F06Rik*, *Grtp1*, *Stk10*) as the most likely QTGs residing within five QTL regions. An additional phenotypic analysis confirmed the predictive power of the gene network analysis.

### Transcriptome

The present study identified 13,726 genes expressed in mouse skeletal muscle, which approximately doubles the number reported earlier in a microarray based study [36]. An expansion of the muscle transcriptome was expected based on the recent comparison between the microarray and RNA-Seq methods in brain tissue [37], and illustrates the superior sensitivity of RNA-Seq. This set of data, therefore, provides a benchmark of expression levels of different genes within mouse muscle tissue, something that was not possible to obtain reliably with microarrays because of variation in sensitivity of hybridization among the probes [10].

The procedure of mapping of sequenced transcriptome fragments on to the reference sequence allows a defined number of mismatches [38]. This provision is particularly important for identification of polymorphisms. However, a side effect of it might be a background noise resulting from the mapping of some of the fragments (likely those originating from homologues) to the genes which in fact are not expressed in the tissue. It is possible to assess the level of such noise empirically by examining expression of the Y chromosome genes in females. The highest level of expression of these genes found in females indicates the threshold below which a reliable assessment of transcript abundance is not possible. Application of this threshold reduced the estimated number of expressed genes by >30%.

A plethora of mouse models divergently selected for various phenotypes have been generated over the decades. These models captured increasing and decreasing alleles of relevant genes and provide a rich resource for studying of the mechanisms underlying variation in specific trait. However, as the genomic sequence of many of these strains is not available yet, utilization of these resources has been hampered. We demonstrated here that the high throughput transcriptome analysis by RNA-Seq provides an effective way for utilizing the potential of selected strains.

### Validation of network analysis prediction

The expression network analysis and integration of the information from the independent datasets provides additional leverage for prioritization of the candidate genes for further interrogation. However, it is important to assess the reliability of prediction. The analyses of gene expression data in rodent muscles indicated that expression pattern of a number of genes in the TA muscle of the SM/J strain is indicative of a more oxidative phenotype compared that of the LG/J strain. Initially this appeared to conflict with our results obtained in the soleus muscle of these strains [21]. The SM/J strain exhibited lower proportion of oxidative type 1 fibers compared to the LG/J strain (e.g., in females 41% vs 58%, respectively). The examination of the TA muscle however confirmed the predicted prevalence of the oxidative phenotype in the SM/J muscle supporting the predictive power of the network analysis (Figure 3). The reversal of the oxidative profile between the soleus and TA muscles could be explained by the distinct composition of muscle fibers constituting these two muscles in mice; soleus is mainly composed of the fibers expressing type 1 and type 2a myosin heavy chains, coded by *Myh7* and *Myh2* genes, respectively. The TA muscle, on the other hand contains very few *Myh7* expressing fibers but is dominated instead by the fibers expressing type 2a, 2x (*Myh1*) and 2b (*Myh4*) myosin heavy chains [39]. From this set of data it appears that the proportion of type 1 *vs* type 2a fibers in the soleus is determined by different mechanisms than the proportion of type 2a, 2x and 2b fibers in TA muscle.

### Candidate genes in the QTL regions

It has been suggested that variation in gene expression is important contributor to the genetic architecture of complex traits [7]. Integration of the gene expression profiling by microarrays and QTL screening in classical mapping populations (backcross or F2) has led to identification of QTGs underlying allergic asthma [8] and bone mineral density [9], and to nomination of the candidate genes underlying adipose tissue [40] just to mention some of the successful attempts to identify QTGs. An improvement in the mapping resolution afforded by an advanced intercross population and enhanced sensitivity of the transcriptome analysis by RNA-Seq provides further incentives for application of this strategy.

Integration of RNA-Seq data with results of QTL mapping from an advanced intercross reduced the number of positional candidates from 1099 genes residing throughout QTL regions to 49 candidate genes differentially expressed or with the coding polymorphisms (with likely functional consequences) between the two strains. These genes were spread across thirteen QTL. Three of those loci, *Skmw25*, *Skmw26* and *Skmw34*, harboured only one candidate gene (Table 1). The candidacy of *Htra2* gene (*Skmw26*) was supported by the *mnd2* model demonstrating a severe muscle wasting phenotype and abnormality of motor neurons resulting from the mutation in the gene [41,42]. The serine peptidase coded by *Htra2* gene is located in the mitochondrial intermembrane space. It activates proapoptotic proteins upon release into the cytosol from damaged mitochondria [43]. Interestingly, in addition to the T449D polymorphism, the transcript abundance of the gene tended to be higher in the SM/J strain (p=0.13; Additional file 1). There is no information on the possible effects of the two genes that are located in single-gene loci (*Osbpl3* in *Skmw25* and *2310014F06Rik* in *Skmw34*). The latter gene is not translated into a protein (http://www.ensembl.org). However, it possesses the properties of the long intergenic non-coding RNA, lincRNA [44], which have been implicated in such biological processes as imprinting [45] and trans-gene regulation [46].

The remaining ten loci (ranging in size from 1.2 Mb to 5.0 Mb) harboured 2 or 3 candidates. From these results it appears that either the trait is truly polygenic, with more than one gene contributing to a QTL even when the latter had been refined to few Mb, or some of these genes are not causative. Validation studies will be required to address this question.

The ability of RNA-Seq to capture splice variants resulted in an interesting candidate gene for *Skmw29* locus. *Irak2* codes for Interleukin-1 receptor-associated kinase 2 which is involved in immune response and is important for activation of NFĸB pathway. Four splice variants of the gene with antagonistic effects were identified in mouse; overexpression of *Irak2a* and *Irak2b* potentiated activation of NFĸB pathway, whereas *Irak2c* and *Irak2d* variants inhibited it [24]. The overexpression of *Irak2* in LG/J strain muscles compared to SM/J strain (Additional file 1) was primarily due to *Irak2c* as the levels of three other variants were similar (Figure 1B). It has been demonstrated that persistent activation of NFĸB pathway caused muscle wasting [47]. Thus, there is a mechanistic link between the levels of expression in *Irak2c* and muscle mass which identifies the gene as a strong candidate to explain the effect of *Skmw29* locus. Overexpression of *Irak2c* might have contributed to larger muscle mass in LG/J strain by inhibiting NFĸB activation.

The *Kdr* gene in the *Skmw24* locus, encodes for one of the vascular endothelial growth factor receptors and is involved in the development of skeletal muscle tissue [48]. Furthermore, it has been shown that an acute response of the skeletal muscle to resistance exercise involves upregulation of its expression [49]. Resistance exercise is a well known muscle mass increasing stimulus, thus it is plausible that the L117F polymorphism in an evolutionarily conserved region might be contributing to the muscle weight difference between the LG/J and SM/J strains.

The gene coding for matrix GLA protein (*Mgp*, *Skmw31*) was shown to be a suppressor of tissue calcification [50]. Mutation of the *MGP* gene in humans causes Keutel syndrome [51]. A higher level of expression of this gene in skeletal muscle was associated with intramuscular fat infiltration known as marbling in cattle [52].

Several of the identified genes are involved in cell signalling (e.g. the genes coding for the regulatory inhibitor subunit 16B of protein phosphatase 1, *Ppp1r16b*, (*Skmw22*), and serine/threonine-protein kinase 10, *Stk10*, (*Skmw42*)), respond to the growth stimulus (growth hormone regulated TBC protein 1, *Grtp1*[53]) or are involved in regulation of transcription (the *Tfdp1* gene encodes for a transcription factor involved in regulation of the cell cycle [54]). Thus, in addition to being differentially expressed or polymorphic these genes also represent the functional candidates which potentially can modify the abundance of muscle tissue.

In addition to the genes discussed above, the *Alpk3* gene in the LG/J strain carries an insertion in exon 5 compared to the SM/J allele. The insertion, CTT, results in additional amino acid, leucine (following the 212 position of the reference sequence), distally of the I-set domain. Functional differences between the two *Alpk3* variants have not been reported.

### QTL lacking candidate genes

Our approach did not suggest any robust candidates for 4 earlier identified QTL. Interestingly, some of those loci had a substantial effect size on muscle mass (i.e. *Skmw23*, *Skmw30*, *Skmw38* and *Skmw41*). Collectively these observations imply that the underlying cause of these loci rest beyond the gene expression patterns in muscle tissue or polymorphisms within the genes. For instance, systemic factors such as hormones can affect muscle tissue. It is also conceivable that the causative genes were expressed during development which might have influenced the number of muscle fibers. In either of those instances no footprint of the influence in muscle transcriptome would be detected.

Only ~4% of differentially expressed genes reside within QTL regions. This observation raises a question about the role of the remaining majority in relation to muscle mass. It can be speculated that secondary changes in gene expression pattern are triggered in the network associated with the QTL causing genes, and genes encoding transcriptional regulators are particularly good candidates. It is also plausible that the systemic factors influencing muscle size are contributing to differential expression between strains. Finally, some of these genes might be involved in other phenotypes and processes which are contrasting between the strains but which are not reflected in muscle weight (e.g. variation in proportion of different fiber types). Integration of the expression data in various tissues at different developmental stages, under different environmental conditions, and profiling of the systemic hormones and growth factors could help understanding of some relationships in gene expression patterns.

## Conclusions

We presented a snapshot of the transcriptome in skeletal muscle from two mouse strains diverging in muscle mass. Furthermore, we showed that overlaying the transcriptome data with the refined genetic architecture of the trait and cross-referencing that with the gene expression network data in skeletal muscle yielded an unbiased list of candidate genes which might affect muscle mass. This integrative approach will facilitate the transition from QTL to detection of their underlying QTGs.

## Materials and methods

The LG/J and SM/J mice (3 males and 3 females of each strain) were purchased from the Jackson laboratory. At the age of 92 days animals were sacrificed and tibialis anterior (TA) muscle was dissected bilaterally, weighed and snap-frozen in isopentane chilled with liquid nitrogen. All procedures were approved by the Institutional Animal Care and Use Committee of the Pennsylvania State University.

### RNA preparation

Total RNA from the TA muscle was extracted using TRIzol (Invitrogen Life Technologies, Carlsbad, CA). Approximately 10 μg of RNA from male and female samples were then submitted for transcriptome sequencing using RNA-Seq technology: SOLEXA/Illumina (The Gene Pool, http//genepool.bio.ed.ac.uk) or HiSeq 2000 (Genome Enterprise Limited; http://www.genome-enterprise.com) platforms.

### RNA-seq

#### Illumina/ SOLEXA GA II

To prepare Illumina RNAseq libraries 3 μg of total RNA were subjected to one round of poly-A selection on oligo(dT) (Serabeads) and resultant mRNA was fragmented to an average size of 100bp following manufacturer’s recommended protocol (Illumina mRNAseq kits Cat no. RS-100-0801). Then, 1st strand cDNA synthesis was carried out using Superscript III reverse transcriptase (Invitrogen cat no.18080051) with the modification of 3μg random primers (Illumina mRNAseq kits Cat no. RS-100-0801). The second strand cDNA and RNAseq libraries were prepared according to the manufacturer’s recommended protocol (Illumina, San Diego, CA, USA). Briefly, the cDNA fragments were blunt ended, A-tailed followed by ligation of Illumina paired end oligo adapters. The adapter ligated fragments were size selected (50 nucleotides) on a 2% agarose gel, and then subjected to 18 cycles of PCR; at this stage modified indexed Illumina primers were used to introduce unique barcodes to each sample. The fragment size, and concentration of resultant libraries were assessed by Qubit (Invitrogen QuantIT HS).), and on a Bioanalyser High Sensitivity Chip. All samples were diluted to 10 nmol in EB/0.1% tween and were de-natured, clustered and sequenced at a density of 1/GA2X lane to yield at least 15M reads/sample.

#### HiSeq2000

The Illumina TruSeq RNA Sample preparation kit (Illumina Inc.) was used according to the manufacturer’s protocol. In brief, poly-A containing mRNA molecules were purified from 0.5 μg total RNA using poly-T oligo attached magnetic beads using two rounds of purification. The purified mRNA was fragmented by addition of 5x fragmentation buffer (Illumina, Hayward, CA) and was heated at 94°C in a thermocycler for 8 minutes. The fragmentation yields fragments of ~250 bp. First strand cDNA was synthesised using random hexamers to eliminate the general bias towards the 3’ end of the transcripts. Second strand cDNA synthesis was done by adding GEX second strand buffer (Illumina, Hayward, CA), dNTPs, RNaseH and DNA polymerase I followed by incubation for 2.5 h at 16°C. Second strand cDNA was further subjected to end repair, A-tailing, and adapter ligation with barcoded adapters in accordance with the manufacturer supplied protocols. Purified cDNA templates were enriched by 15 cycles of PCR for 10 s at 98°C, 30 s at 60°C, and 30 s at 72°C using PCR Primer Mix Cocktail and PCR Master Mix (Illumina, Hayward, CA). The samples were cleaned using AMPure XP Beads and eluted in 30 μl Resuspension Buffer as per manufacturer's instructions (QIAGEN, CA). Purified cDNA libraries were quantified using Bioanalyzer DNA 100 Chip (Agilent Technology 2100 Bioanalyzer). The libraries were normalised to 10 nM and pooled equimolarly in pools of 2 samples per pool.

### Bioinformatics

Sequencing reads were aligned to the reference mouse genome (UCSC assembly mm9) using tophat v 1.3.1 [38] allowing 1 alignment per read and mapping to known exon-exon junctions of known Ensemble genes. The number of reads mapping to each Ensemble gene was counted with htseq (http://www-huber.embl.de/users/anders/HTSeq/doc/overview.html). Statistical analysis was performed in R using the bioconductor package Deseq, based on the negative binomial distribution, with variance and mean linked by local regression [55] and baySeq, which uses an empirical Bayes approach [56]. Variant analysis was performed with samtools 0.1.14 [57], annotation of variants was performed with seqgene v 2.3 [58]. SNPs and Indels with Variant and Mapping quality >20 and present in all replicate samples were marked as potentially significant. Dexseq was used for analysis of differentially expressed exons, visualization and exploration for identification of differentially expressed splice variants [59]. To overcome some of the limitations of DexSeq with respect to correct identification of all differentially expressed exon-bins when many exon-bins in one gene model are affected, we used both DexSeq statistics and visualisation of normalised counts and, in addition, we calculated strain mean and fold change between strains for interpretation of the results as exemplified for *Irak2* (Table 2).

The PolyPhen web based tool [60] was utilized to predict the possible effects of amino acid substitution on the function of a protein (http://genetics.bwh.harvard.edu/pph/). These predictions are based on multiple sequence alignments, and functional and structural characterization of the substitution site.

### RT PCR

Expression of splice variants of the *Irak2* gene were examined using combinations of Irak2-1 (forward, F) and Irak2-3 (reverse, R; for *Irak2a* and *Irak2b* variants), and Irak2-7 (F), Irak2-3 (R; for *Irak2d*) primers reported by Hardy and O’Neill [24]; and a pair of primers for Irak2c (F, 5’-GCACTGACTGAGGGAAAAGG-3’; R, 5’-CCAAAAGCCTTTCTTGCTTG-3’).

The RT PCR procedure has been described in detail [61]. The images of the PCR products were quantified using ImageJ software (NIH – version 1.43).

### F2 cross data for network analyses

Six of the nine crosses reported in this paper (BTBRxB6ob, BxH, BxHapoe, BxA_JaxS, BxA_MCI and BxD_PSU) have been previously published [2,27,28,29,30]. Of the remaining three crosses, two (Bx129_JaxS and BxD_JaxS) are from the same experimental design as BxA_JaxS but are from Bx129 and BxD backgrounds, respectively. The third cross, BxD_JaxL, is also a BxD background but rather than a 20 week design (JaxS) the mice were aged for 64 weeks (JaxL).

### Statistical analyses

Unless otherwise stated, statistical comparisons were carried out using a t-test, and the data presented as mean ± SD.

## Competing interests

The authors declare that they have no competing interests.

## Authors' contributions

AL conceived the study, collected phenotypes, did molecular biology and drafted the manuscript; CM carried out bioinformatics including sequence alignment, screening for SNPs, indles and splice variants and helped to draft the manuscript; JMJD made substantial contribution to design of the study, conducted network analysis, and drafted the manuscript; AR carried out molecular biology and helped drafting the manuscript; AMC carried out histology and participated in drafting of the manuscript; DJV participated in preparation of the manuscript; DAB collected phenotypes and helped drafting the manuscript. All authors read and approved the final manuscript.

## Supplementary Material

Additional file 1 Contains RNA-Seq results. Click here for file

Additional file 2 **Contains scatter plot of the transcriptome of the TA muscle.** X axis, mean expression level, Y axis, log of fold difference between LG/J and SM/J strains. Red dots represent differentially expressed genes at p<0.1. Click here for file

Additional file 3 Contains DexSeq analysis results. Click here for file

Additional file 4 Contains identified SNPs. Click here for file

Additional file 5 Contains identified Indels. Click here for file

Additional file 6 Contains genes of Mouse Muscle Bayesian Network (MMBN). Click here for file

Additional file 7 Contains Key Driver network analysis results. Click here for file

Additional file 8 Contains DAVID analyses results in DE and LSRN gene sets. Click here for file
