# Supplementary figures and images for: Resolving candidate genes of mouse skeletal muscle QTL via RNA-Seq and expression network analyses
Source: BMC Genomics. 2012 Nov 5;13:592. doi: 10.1186/1471-2164-13-592 (PMC3505184; doi:10.1186/1471-2164-13-592)

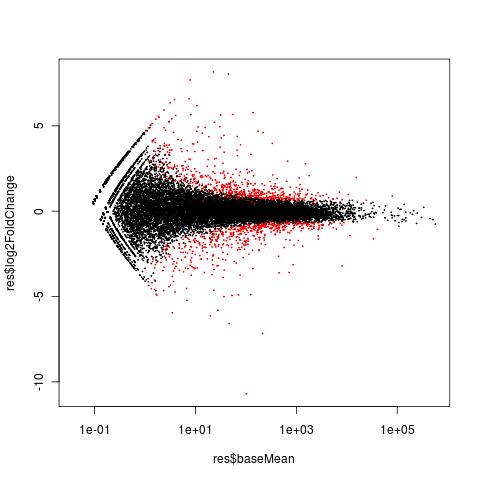

Supplement: Additional file 2 — Contains scatter plot of the transcriptome of the TA muscle. X axis, mean expression level, Y axis, log of fold difference between LG/J and SM/J strains. Red dots represent differentially expressed genes at p<0.1. [file 1471-2164-13-592-S2.jpeg]
